# Genealogy of Instruments for Prodrome Evaluation of Psychosis

**DOI:** 10.3389/fpsyt.2013.00025

**Published:** 2013-04-18

**Authors:** Jean-Gabriel Daneault, Emmanuel Stip, Refer-O-Scope Group

**Affiliations:** ^1^Département de Psychiatrie, Université de MontréalMontréal, QC, Canada; ^2^Centre de Recherche Fernand-Séguin, Université de MontréalMontréal, QC, Canada

**Keywords:** psychosis, prodrome, at risk mental state, ultra-high risk state, prediction and forecasting, review of literature

## Abstract

**Objective**: Over the last 15 years, researchers from around the world have developed instruments for assessing the risk of conversion to psychosis. The objective of this article is to review the literature on these instruments by focusing on genealogy links and on their performance in predicting conversion to psychosis.

**Method:** A systematic review of articles published since 1980 relating to risk assessment instruments for conversion to psychosis by manual search and consultation of electronic databases MEDLINE, EMBASE, and PsycINFO.

**Results:** Three hundred ninety one (391) publications were selected and analyzed. Among these, 22 instruments were identified. These instruments are briefly described and placed on a timeline according to their year of publication. A code of positions, patterns, and forms is used to schematize the characteristics of each instrument. A table is presented to show changes in rates of conversion to psychosis within cohorts of subjects considered at risk according to the instruments. A second code of shades and outlines is used to schematize the characteristics of each cohort of patients. The two graphics set the stage for a discussion about the major strategies that were adopted to improve the performance of risk assessment instruments.

**Conclusion:** These graphics allow a better understanding of the origin, evolution, current status, strengths, shortcomings, and future prospects of research on risk assessment instruments.

**Clinical Implications**

- The integration of theoretical approaches, the multicenter studies, and the pre-selection of patients with short questionnaires were the main strategies to improve the performance of instruments assessing the risk of conversion to psychosis.
- These instruments are better at predicting conversion to psychosis than conventional variables within a more limited time span and can therefore enable the evaluation of various risk factors and biomarkers that may be associated with psychosis.

**Limitations**

- The studies selected for this review of literature were not classified according to their methodological quality.
- These studies are based on heterogeneous populations and this must be taken into account when comparing the rates of conversion to psychosis.
- This review of literature was based on published data only and they were no direct communication with the authors of these instruments.

## Introduction

Beyond its economic implications, psychosis is a disease that all too often has a lasting and painful impact on the patient’s life. In the hope of avoiding the most dramatic consequences, research groups around the world have been working on psychosis prevention since the early 1990s (McGlashan and Johannessen, 1996).

Literature reviews show that a longer duration of untreated psychosis is associated with higher scores for overall psychopathology, positive symptoms and negative symptoms, and with a lower level of functioning (Marshall et al., 2005; Perkins et al., 2005). This observation has led to the creation of early intervention programs that aim to reduce the duration of untreated psychosis in order to ward off the disorder’s toxic effects on the brain.

Recently, the results of the TIPS, OPUS, and LEO studies have contributed to the development of knowledge concerning early intervention programs. In Scandinavia, Larsen et al. (2011) showed that patients who had a shorter duration of untreated psychosis achieved better results on scales of social functioning and negative, depressive, and cognitive symptoms after 5 years of follow-up. The OPUS study in Denmark and the LEO study in the UK showed the beneficial effects of intensive treatment for patients experiencing a first psychotic episode, after 2 years and after 18 months, respectively (Craig et al., 2004; Petersen et al., 2005). Unfortunately, these beneficial effects were not maintained after 5 years of follow-up (Bertelsen et al., 2008; Gafoor et al., 2010).

In light of these results, it is possible that the duration of untreated psychosis is actually a marker of a more severe disease, rather than a modifiable determinant of the course of the disease (McGlashan, 1999; Bosanac et al., 2010). Alternatively, it has been suggested that even earlier interventions, before the psychosis manifests itself, could change the course of the disease (McGlashan and Johannessen, 1996).

In point of fact, an appreciable proportion of patients who have experienced a psychotic episode will find it difficult to work and socialize even if they have received early treatment and have experienced a short duration of untreated psychosis (Addington et al., 2003). This kind of disrupted functioning seems to develop in the period preceding the onset of psychosis (Hafner et al., 1999).

If we define conversion to psychosis as the transition from a status without a psychotic disorder to a diagnosis of a psychotic disorder (according to the clinical assessment or based on psychometric scales), the development of instruments to evaluate the risk of conversion to psychosis would make it possible to identify individuals who will develop psychosis before the disease sets in and therefore before their lives are disrupted. These people could then receive treatments to mitigate, delay, or even prevent the undesirable consequences. For more than 20 years, such instruments have been developed and tested around the world, starting with the scales created by Chapman and Chapman (1987), who sought to identify individuals at risk of conversion to psychosis beyond the genetic risk represented by the existence of a close relative with schizophrenia or an affective disorder with psychotic characteristics.

However, the conversion rate in the cohorts of individuals identified as being at risk of developing psychosis are relatively low, ranging from less than 10% to slightly over 50% within follow-up periods ranging from 6 months to 10 years. In many centers, it has also been observed that conversion rates decline with time (Yung et al., 2007). In this context, the identification of individuals who are at risk of conversion to psychosis itself poses a risk of stigmatization, which is all the more problematic given that a significant proportion of these people will not actually develop psychosis. The idea of testing interventions on these individuals is therefore a daunting one, because the interventions themselves have a considerable risk of undesirable side effects (Cornblatt et al., 2001).

In the face of these obstacles and ethical issues, research teams have sought to improve the performance of their instruments. Instruments for evaluating the risk of conversion to psychosis have proliferated and it has become complicated to determine the genealogy of each of these instruments. Where does a particular instrument come from? How is it related to a specific older or newer instrument? And how well does it perform in predicting psychosis? Based on schematic representations, this article aims to answer these questions and help readers to find their way in a fast-changing literature and to consider the future of research on prodromal psychosis.

## Methods

To do this, a review of the literature on instruments designed to evaluate the risk of conversion to psychosis was done by our group from November 2009 to July 2011. The keywords *psychosis*, *prodrome*, *prodromal*, *at-risk mental state*, *ultra-high risk state*, *prediction and forecasting*, *prospective study*, *schizophrenia and disorders with psychotic features*, *preventive psychiatry* and *affective disorders, psychotic* were combined in the following search engines: PsycINFO 1967–July week 2 2011, EMBASE 1980–2011 week 29, and Ovid MEDLINE^®^ 1948–July week 2, 2011. Additional publications were obtained by a manual search of the references of the publications that were found initially. These publications were read and annotated by at least three of the authors (Danièle Blais, Jean-Gabriel Daneault, and Emmanuel Stip). The publications retained had to concern one or more instruments for evaluating the risk of conversion to psychosis and to be written in English or French. Particular attention was paid to previously published literature reviews on these instruments to better understand their theoretical bases.

**Figure 1:**
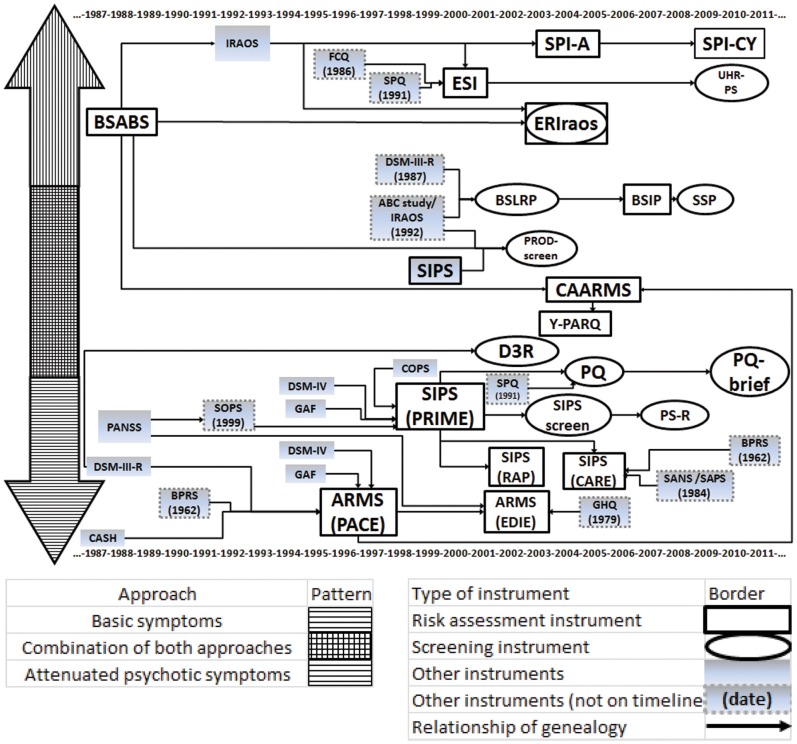
**Timeline of instruments assessing the risk of conversion to psychosis**. BSABS, Bonn scale for the assessment of basic symptoms; IRAOS, interview for the retrospective assessment of the onset and course of schizophrenia and other psychosis; FCQ, Frankfurt complaint questionnaire; SPQ, schizotypal personality questionnaire; ESI, Eppendorf schizophrenia inventory; SPI-A, schizophrenia prediction instrument – adult version; SPI-CY, schizophrenia prediction instrument – child and youth version; UHR-PS, ultra high risk psychosis scale; ERIraos, early recognition inventory for the retrospective assessment of the onset of schizophrenia; DSM-III-R, diagnostic and statistical manual of mental disorders – third edition – revised; ABC Study, age, beginning, and course study; BSLRP, Basel screening list for risk of psychosis; BSIP, Basel screening instrument for psychosis; SSP, self-screen prodrome; CAARMS, comprehensive assessment of at-risk mental states; Y-PARQ, youth psychosis at risk questionnaire; PANSS, positive and negative syndrome scale; SOPS, scale of prodromal symptoms; CASH, comprehensive assessment of symptoms and history; BPRS, brief psychiatric rating scale; GAF Scale, global assessment of functioning scale; ARMS, at-risk mental state; PACE clinic, personal assistance and crisis evaluation clinic; COPS, criteria for prodromal states; SIPS, structured interview for prodromal symptoms; PRIME Clinic, prevention via risk identification, management, and education clinic; D3R, a selection of three of the criteria of prodromal schizophrenia according to the DSM-III-R; SPQ, schizotypal personality questionnaire; PQ, prodromal questionnaire; PS-revised, PRIME-screen revised; EDIE Study, early detection and intervention evaluation study; GHQ, general health questionnaire; RAP, recognition and prevention program; CARE program, cognitive assessment and risk evaluation program; SANS, scale for the assessment of negative symptoms; SAPS, scale for the assessment of positive symptoms.

The scales and other tools on which they were based were placed on a timeline (Figure 1), and classified with a pattern code. The genealogical relations were represented with single arrows. A table was prepared to show the change over time in the rates of conversion to psychosis within the cohorts of subjects considered to be at risk (Table 1). The publications retained for this table had to concern samples of patients identified as being at risk of conversion to psychosis by one or more evaluation instruments, who had been followed up prospectively for at least 6 months and on whom sociodemographic information was provided (at least the mean age and percentages of men). Although most of these cohorts underwent more intensive clinical follow-up (and thus some kind of intervention), the publications that concerned more formal treatments (such as a series of psychotherapy sessions or a drug trial) were set aside. When a single cohort was the subject of several publications, only the most recent one was retained. A second code involving shades of gray and border styles was established to enable readers to distinguish the main characteristics of each cohort.

**Table 1:** **Changes in rates of conversion to psychosis within cohorts of subjects considered at risk**.

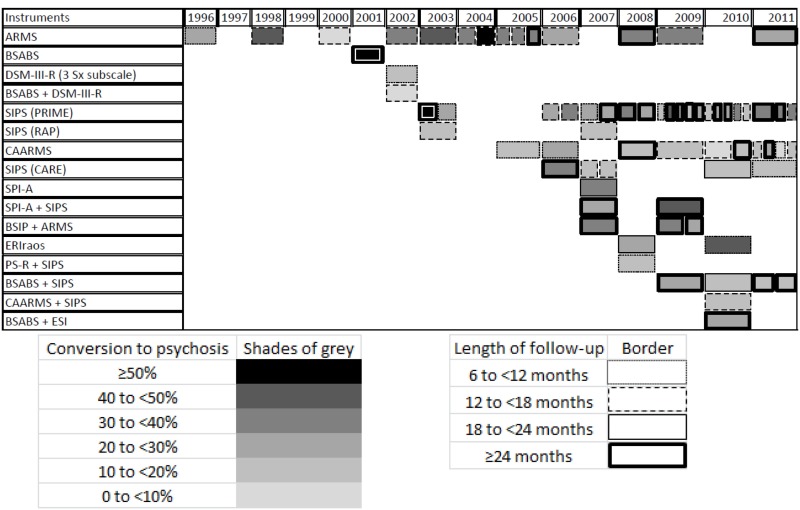

*BSABS, Bonn scale for the assessment of basic symptoms; ARMS, at-risk mental state; DSM-III-R, diagnostic and statistical manual of mental disorders – third edition – revised; BSABS + DSM-III-R, combination of BSABS and DSM-III-R; SIPS, structured interview for prodromal symptoms; PRIME clinic, prevention via risk identification, management and education clinic; RAP, recognition and prevention program; CAARMS, comprehensive assessment of at-risk mental states; CARE program, cognitive assessment and risk evaluation program; SPI-A, schizophrenia prediction instrument – adult version; BSIP, Basel screening instrument for psychosis; ERIraos, early recognition inventory for the retrospective assessment of the onset of schizophrenia; PS-revised, PRIME-screen revised; ESI, Eppendorf schizophrenia inventory*.

## Results

The search in PsycINFO returned 240 publications, in EMBASE, 249, and in MEDLINE, 63. After adding the publications found with a manual search and applying the inclusion and exclusion criteria, we retained a total of 391 publications. Of this number, 181 publications (46.3%) came from Europe, 123 (31.5%) from North America, 51 (13.0%) from Australia, 14 (3.6%) from Asia, and 2 (0.5%) from Israel. Certain publications featured contributions by authors from several research centers around the world, and 20 (5.1%) were classified as the outcome of a multicenter cooperative study. Several of these publications concerned the follow-up of cohorts of patients at risk of conversion to psychosis (155 or 39.6%). As well, 71 reviews of the literature (or 18.2% of the publications retained) enabled us to consolidate our knowledge of the topic.

Through this search, we identified 22 instruments for evaluation of the risk of conversion to psychosis. These instruments were placed on the timeline based on their publication dates (see Figure 1). Like Olsen and Rosenbaum (2006), we first classified them according to two major approaches that predominate in the research on the prodrome of psychosis. The instruments that pay most attention to attenuated psychotic symptoms were placed below the timeline. They aim to identify individuals at risk of imminent conversion to psychosis. It should be recalled that attenuated psychotic symptoms are not risk factors as such. Rather, they constitute what are generally called precursors of the disease (Eaton et al., 1995).

The instruments that instead focus on basic symptoms were placed in the upper half of the figure. The concept of basic symptoms refers to the first phase of subjective disturbance (in which the subject’s experience of volition, emotions, thoughts, language, sensory perceptions, and motor actions becomes different or abnormal), which occurs at the very start of the development of psychosis (Ruhrmann et al., 2010). In addition to these two major schools of thought, there is a third category of instruments comprising tools that combine theoretical concepts from both approaches. Thus, the closer an instrument is to the center of the figure, the more it incorporates elements from both schools of thought. Our comparison of the instruments also allowed us to identify two different types of tools: structured or semi-structured interviews that require the presence of specialized investigators (placed in a rectangle) and shorter questionnaires that may be self-administered (in an oval).

Developed in Germany, the *BSABS* was designed to assess the major categories of basic symptoms: “dynamic deficiencies” (including emotional, perceptual, and action disorders), cognitive problems, disorders affecting cenesthesia (the sense of bodily existence), central neurovegetative disorders, and coping strategies (Gross et al., 1987). In the Cologne Early Recognition project, 70% of subjects considered at risk by the BSABS developed schizophrenia after 9.6 years of follow-up (Klosterkotter et al., 2001). We have not found any other publications that used the BSABS alone and in its original version to follow-up subjects at risk of conversion to psychosis.

The *ERIraos* is an instrument composed of the 10 BSABS items with the greatest predictive validity for psychosis; it also incorporates the findings of the ABC Study on the period preceding the onset of psychosis, based on a retrospective evaluation conducted with a semi-structured interview, the IRAOS (Hafner et al., 1998). The evaluation takes place in two sessions. Subjects are invited to undergo an interview on the basis of their scores on a screening questionnaire, which may be self-administered (Hafner et al., 2004). In the German Research Network on Schizophrenia, Hurlemann et al. (2008) found that 22.2% of a group of subjects identified as at risk by the ERIraos had developed psychosis at the end of an 18-month follow-up. Another cohort deemed at risk by the ERIraos showed a 40.3% conversion rate after a median follow-up period of 32 months (Bodatsch et al., 2011).

The *SPI-A* was developed further to a facet- and data-cluster-based analysis of the basic symptoms reported by subjects at risk of conversion to psychosis in the Cologne Early Recognition project (Schultze-Lutter et al., 2004, 2007). The SPI-A was used by itself in a publication that showed that 34.9% of a group of subjects considered to be at risk presented a psychotic illness at the end of a 20.6-month follow-up period (Schultze-Lutter et al., 2007). When the SPI-A was used together with SIPS, the conversion rate was 29% at the end of 3 years (Huang et al., 2007) and 41% at the end of 5.1 years of follow-up (Koethe et al., 2009).

Since the results of these analyses were very different in children and adolescents, a version for subjects aged 8- to 18-years-old was developed at the University of Heidelberg. This instrument, the *SPI-CY* emphasizes the distinction between basic symptoms and other clinical pictures in child psychiatry and incorporates parents’ observations (Koch et al., 2007). At the time of writing, no publication had yet reported rates of conversion to psychosis associated with the SPI-CY.

The criteria for an at-risk mental state (*ARMS*) were first operationalized by the research team at the PACE Clinic in Melbourne, VIC, Australia. They refer to three groups of patients: patients who present attenuated psychotic symptoms (i.e., symptoms that are significantly different from normal without reaching psychotic intensity, according to the CASH and the BPRS); patients who present what are known as Brief Limited Intermittent Psychotic Symptoms or BLIPS (i.e., symptoms at the psychotic level of intensity according to the CASH and BPRS, but that clear up spontaneously within less than a week); and patients who present risk factors combined with an at-risk state or *trait and state risk factors* [i.e., subjects between the ages of 14 and 30 who present a schizotypal personality or a family background of psychotic disorders as defined by the DSM-IV (Diagnostic and Statistical Manual of Mental Disorders – Fourth Edition), with sufficient disturbance of their mental state to decrease their score on the Global Assessment of Functioning (GAF) scale by at least 30 points] (McGorry et al., 1996; Yung et al., 1996). The conversion rates associated with the ARMS criteria have remained relatively stable (or with a slight downward trend) over time (e.g., at the PACE Clinic, after 12 months of follow-up, the rate was 48% in 1998 vs. 33.3% in 2002 vs. 34.3% in 2009) (Yung et al., 1998; Phillips et al., 2002; Sun et al., 2009).

The *EDIE* Study also applies these criteria, but it does not use the CASH and the BPRS, defining the level of psychotic intensity by means of the PANSS instead. A decline of at least 30 points on the GAF is still considered to be a criterion for an at-risk state, but it may also be defined by a positive score on the GHQ (Morrison et al., 2002). The EDIE Study is a randomized controlled study in which 35 subjects considered to be at risk of conversion received 6 months of cognitive therapy, while 23 other subjects also considered to be at risk received regular follow-up. After 36 months, conversion rates were 20 and 22%, respectively (not a statistically significant difference) (Morrison et al., 2007). Given that this was a randomized controlled study, this result has not been retained for the remainder of the discussion.

In addition to assessing the criteria for ARMS, as defined above, the semi-structured interview in the *CAARMS* makes it possible to investigate basic symptoms (Yung et al., 2005). We can see a downward trend in the conversion rates associated with the CAARMS. Lam et al. (2006) reported a 29% conversion rate after 6 months of follow-up, whereas Yung et al. (2008) found a rate of 16% after 24 months, Demjaha et al.’s (2012) rate was 14.8% after 24 months; finally, Nelson et al.’s (2011) study had a rate of 8.8% after 6 months.

The *Y-PARQ* is an instrument with 92 items to which the respondent can answer “yes,” “no,” or “unknown.” It was based on the CAARMS and was developed for the PEPS study, which investigated teens on Palau, a Pacific island where there is a high frequency of familial schizophrenia (Ord et al., 2004; Myles-Worsley et al., 2007a,b). At the time of writing, no publication had yet reported rates of conversion to psychosis associated with the Y-PARQ.

The *SIPS* is a semi-structured interview developed by Miller and McGlashan at the PRIME clinic in New Haven, Connecticut, to evaluate the severity of ARMS, as defined by Yung and McGorry. It is made up of the SOPS, which is modeled on the PANSS, and of the GAF, the criteria for schizotypal personality disorder according to the DSM-IV, and the COPS (Miller et al., 1999). It is not possible to detect any clear downward or upward trend when we analyze changes in the conversion rates associated with the SIPS. These conversion rates range from 13.5% after a 12-month follow-up (Simon and Umbricht, 2010) to 57.1% after 24 months (Miller et al., 2003).

The SIPS was used by the *RAP* program at Zucker Hillside Hospital in New York to define Clinical High Risk (CHR). According to this research strategy, patients are at risk of conversion to psychosis if they present attenuated positive symptoms (or CHR+), but also if they manifest attenuated negative symptoms (or CHR−) (Cornblatt et al., 2003). In a publication from 2003, 14.5% of subjects identified as being at risk of conversion developed psychosis after approximately 12 months (Cornblatt et al., 2003). In another cohort, 19% of subjects presented a psychotic illness after 12 months’ follow-up (Cornblatt et al., 2007).

The SIPS is also used by the *CARE* program at the University of San Diego. Subjects considered to be at risk of conversion go through a battery of tests including the SIPS, the SANS, the SAPS, and the BPRS, to better take into account the negative symptoms and disorganized behaviors into risk assessment (Haroun et al., 2006). There seems to be a downward trend in the conversion rates associated with the SIPS as used in the CARE program. Haroun et al. (2006) reported that 30% of subjects considered to be at risk had developed psychosis after 3 years of follow-up. However, in a 2010 publication, only 12.5% of subjects considered to be at risk had developed a psychotic illness. Nevertheless, this reduced rate must be interpreted in the context of a shorter follow-up period, 18 months (Jahshan et al., 2010).

The *PRIME-screening test* or *PRIME-screen* or *SIPS-screen* is composed of 12 items from the SIPS. It is a screening test that can be self-administered in front-line clinics (Miller et al., 2004). A modified version, the *PS-revised* (or PRIME-screen revised), was developed by a Japanese team that added a section on the duration of symptoms to improve its specificity (symptoms present for a longer time are considered to be more representative of being at risk of conversion to psychosis) (Kobayashi et al., 2008; Morita et al., 2010). Together with the SIPS, this screening test was tested on a population of 35 subjects identified as being at risk of conversion to psychosis; 14.8% of them developed psychosis after 6 months (Kobayashi et al., 2008).

Inspired by the SIPS and the SPQ, the *PQ* is a self-evaluation screening instrument made up of 92 “true or false” statements. Created at the University of California, Los Angeles, it seeks to identify subjects who are potentially at risk of conversion to psychosis before they undergo a more formal risk assessment interview (Loewy et al., 2005). The *PQ-brief* is a modified version of the original PQ. Its 21 items are essentially the outcome of the selection and revision of statements concerning positive symptoms (Loewy et al., 2011). At the time of writing, no publication had reported rates of conversion to psychosis associated with the PQ or PQ-brief.

The *ESI* is a 40-item questionnaire that attempts to evaluate disorders affecting psychotic patients’ experience of cognitive functions (Mass et al., 2005). It is inspired by the BSABS, the FCQ (Sullwold and Huber, 1986), and the SPQ (Raine, 1991). Niessen et al. (2010) used it in a sample that included subjects who requested psychological help and patients with a mental illness. The ESI proved to be able to distinguish between patients with active psychosis and subjects at risk of conversion. When used with the BSABS and the SIPS, the ESI was able to identify 64 subjects considered to be at risk of conversion. After 36 months’ follow-up, 20.3% of these subjects had developed a psychotic illness (Niessen et al., 2010). A selection of five items from the ESI led to the creation of the *UHR-PS*, which could potentially make it easier to screen at risk patients (Niessen et al., 2010).

The *DSM-III-R* also suggests criteria for identifying prodromal schizophrenia. These criteria are relatively unspecific, especially in the adolescent population (McGorry et al., 1995). On the other hand, Horneland et al. (2002) found that three of the criteria (“peculiar behavior,” “magical thinking,” and “unusual perceptual experiences” – *D3R*) more specifically indicate an increased risk of conversion to psychosis. In a sample of 501 subjects with at least one DSM-III-R criterion for prodrome, the D3R identified 20 subjects as being at risk of conversion to psychosis; 3 of them (15%) developed a psychosis after 6 months of follow-up (Horneland et al., 2002).

The FEPSY-Projekt (or Basel early detection of psychosis study) in Switzerland led to the development of several instruments inspired by the DSM-III-R criteria, but also by the literature on prodromal psychosis (with the use of data obtained in the ABC Study, among other things). The *BSLRP* is a short screening questionnaire designed to help general physicians make decisions about referring potentially at risk subjects for more specialized services (Asston et al., 2002; Gschwandtner et al., 2003). The *BSIP* includes 46 items, but is intended for psychiatrists working with a population that has requested mental health assistance (Riecher-Rossler et al., 2007). The *SSP* is a self-evaluation screening tool made up of 32 true or false questions (Mueller et al., 2009; Muller et al., 2010). The conversion rates associated with the BSIP show the following trend: used in conjunction with the ARMS criteria, 34% conversion to psychosis after 25 months (Borgwardt et al., 2007), likewise 34% but after 5.4 years (Riecher-Rössler et al., 2009) and 28.6% after 6 years of follow-up (Gschwandtner et al., 2009).

The *PROD-screen* is a 29-question screening questionnaire inspired by the SIPS, the IRAOS, and the BSABS (Heinimaa et al., 2003). Because of its concise and simple format, it can be self-administered or administered over the telephone to identify subjects who should be evaluated in more depth with a semi-structured interview. At the time of writing, there had not yet been any publications reporting the rate of conversion to psychosis associated with the PROD-screen.

Most of these instruments were tested on cohorts of subjects considered to be at risk of conversion to psychosis. Table 1 shows the changes in the rates of conversion to psychosis over time, as a function of the instrument used.

## Discussion

One of the first observations that emerge from the analysis of Figure 1 is that the timeline is loaded with a large number of instruments. These various instruments are the outcome of repeated revisions of earlier instruments on the basis of statistical analyses (which have often sought, among other things, to determine the criteria that best predict conversion to psychosis) and based on the clinical experience of the researchers involved and the obstacles they encountered.

Faced with rather low rates of conversion to psychosis, several research teams have attempted to combine the two major approaches for evaluating prodromal psychosis (i.e., the approach based on attenuated psychotic symptoms and the one based on basic symptoms). This is well represented in Figure 1 by the appearance over time of instruments such as the CAARMS and the PROD-screen, which have been placed in the center of the diagram to make their theoretical foundations clear. This is also shown in Table 1, where one can clearly see that research teams are investing more and more time in combining evaluation tools so they can make use of both approaches. It is postulated that this combination will make it possible to identify the groups of subjects who are most representative of patients who will develop psychosis (Simon et al., 2006).

But this reconciliation of the two approaches is not sufficient to mitigate the differences between the various research teams. Differences remain between the characteristics of the basic population, the recruitment of patients, the evaluation process, the follow-up, the treatments provided, etc. This is why there was the creation of several multicenter studies (the PRIME project, the NAPLS, and the EPOS). In addition to enabling researchers to test evaluation instruments on a much larger population, these studies are pursuing the homogenization of the process of evaluating subjects at risk of conversion to psychosis. This homogenization makes it easier to compare the results of research on these subjects, which had formerly been difficult and risky.

Figure 1 also shows that questionnaires to facilitate screening have proliferated in recent years. In this regard, it is important to remember that the first publications on the evaluation of the risk of conversion to psychosis concerned very well-selected cohorts of subjects. Those subjects were usually referred by mental health professionals who already suspected that a psychotic process was developing. In this context, it is crucial to develop shorter instruments that can be completed in the form of a self-evaluation or a telephone interview by a non-specialist evaluator to separate out the subjects who need an in-depth interview with a specialist in prodromal psychosis and the subjects who have non-specific complaints that can be handled by first-line services. It has in fact been shown that patients are quite reliable at reporting psychotic-type symptoms, as indicated by the good match between self-evaluations and evaluations done by specialized observers (Lincoln et al., 2010).

We should point out that the way subjects get into programs for evaluating the risk of conversion probably has a great influence on the proportion of subjects who will develop psychosis out of the total cohort of patients considered to be at risk. Yung et al. (2007) hypothesized that quicker referral could explain the decline in conversion rates observed in recent years by several screening programs for early psychosis (Yung et al., 2007). This aspect is well represented in Table 1, where the table fields are increasingly likely to be pale gray (indicating low conversion rates of approximately 0–20%) as time passes, despite the fact that their borders become thicker and darker (indicating that follow-up periods are longer, up to 2 years or more).

Despite their relatively low positive predictive value, it should be emphasized that these evaluation instruments are better at predicting conversion to psychosis than conventional variables (e.g., family background of psychotic disorders) within a more limited time span (Cannon, 2008). They make it possible to identify cohorts in which 10–50% of individuals will develop psychosis after approximately 2–3 years of follow-up. These cohorts are therefore fertile ground for evaluating the predictive value of various risk factors and biomarkers that may be associated with conversion to psychosis. As an example, Thompson et al. (2011) reported that three clinical variables that enhanced the predictive validity of an eventual conversion to psychosis (i.e., family history of psychosis with functional decline, high scores for unusual thought content, and poor overall level of functioning) in a sample of at risk patients studied by the NAPLS consortium also significantly improved the predictive validity of an eventual conversion to psychosis in a sample of at risk patients studied at the PACE clinic in Melbourne, VIC, Australia. These cohorts should also make it easier to study the onset of clinical manifestations of psychosis. A better understanding of the first signs and symptoms of the disease could eventually enable clinicians to make earlier and more solidly based diagnoses.

## Conclusion

These results show how these instruments are evolving. They are constantly being revised as a function of the clinical and ethical challenges represented by research on the prodrome of psychosis. This is what we have attempted to schematize in Figure 1. One limitation on Figure 1 is that, although it clearly shows the genealogical relations between the different instruments, it may not make it sufficiently clear that some advances are based on the generation of new tools in an essentially spontaneous and intuitive way. The creation of this figure also led us to examine the variations in these instruments’ performance. We represented these variations in Table 1 in the form of conversion rates to psychosis within cohorts of patients considered to be at risk. This presentation is limited by the lack of information on each instrument’s sensitivity and specificity. Unfortunately, most of the publications examined present only a group of subjects considered to be at risk of conversion to psychosis (without comparison with a control group of subjects considered to have little or no risk of conversion). Thus, it was not always possible to calculate (and present) these parameters. Another limitation is the lack of information provided about the subjects’ origins or the specific features of their referral for assessment of the risk of conversion to psychosis. Several of the publications simply mention that the subjects were help-seekers who were seen by front-line health care professionals and then referred to general mental health clinics or more specialized clinics due to a suspicion that they were experiencing the onset of a psychotic illness. Given that epidemiological studies have shown that, in the general population, the experience of psychotic symptoms may be relatively frequent (approximately 5%), and that they are generally transitory (van Os et al., 2009), it appears probable that, beyond the quality of the evaluations done with the various instruments, the way in which the subjects were selected is also important. It is possible that the groups were very different in terms of the subjects’ origins and how they were referred to the evaluators. It is also possible that such differences explain some of the variation in conversion rates at the follow-up sessions.

One of the next steps could be to push the analysis further and to consider changes in these patients’ distress levels and functioning. As Ruhrmann et al. (2010) emphasize, it has been shown that a high proportion of these patients have to cope with disrupted social and occupational functioning and deterioration in their quality of life, regardless of whether or not they convert to psychosis (Ruhrmann et al., 2010). Instruments for evaluating the risk of conversion to psychosis could also measure symptomatic and functional impairment separately. It would then make it possible to identify these suffering and dysfunctioning patients so that they can be offered the appropriate health care services.

## Conflict of Interest Statement

The authors declare that the research was conducted in the absence of any commercial or financial relationships that could be construed as a potential conflict of interest.

## Abbreviations

ABC Study, age, beginning, and course study; ARMS, at risk mental state; BPRS, brief psychiatric rating scale; BSABS, Bonn scale for the assessment of basic symptoms; BSIP, Basel screening instrument for psychosis; BSLRP, Basel screening list for risk of psychosis; CAARMS, comprehensive assessment of at-risk mental states; CARE program, cognitive assessment and risk evaluation program; CASH, comprehensive assessment of symptoms and history; COPS, criteria for prodromal states; D3R, a selection of three of the criteria of prodromal schizophrenia according to the DSM-III-R; DSM-III-R, diagnostic and statistical manual of mental disorders – third edition – revised; EDIE study, early detection and intervention evaluation study; EPOS, European prediction of psychosis study; ERIraos, early recognition inventory for the retrospective assessment of the onset of schizophrenia; ESI, Eppendorf schizophrenia inventory; FCQ, Frankfurt complaint questionnaire; GAF scale, global assessment of functioning scale; GHQ, general health questionnaire; IRAOS, interview for the retrospective assessment of the onset and course of schizophrenia and other psychosis; NAPLS, North American prodrome longitudinal study; PACE Clinic, personal assistance and crisis evaluation clinic; PANSS, positive and negative syndrome scale; PEPS, Palau early psychosis study; PRIME clinic, prevention via risk identification, management and education clinic; PS-Revised, PRIME-screen revised; PQ, prodromal questionnaire; RAP, recognition and prevention program; SANS, scale for the assessment of negative symptoms; SAPS, scale for the assessment of positive symptoms; SIPS, structured interview for prodromal symptoms; SOPS, scale of prodromal symptoms; SPI-A, schizophrenia prediction instrument – adult version; SPI-CY, schizophrenia prediction instrument – child and youth version; SPQ, schizotypal personality questionnaire; SSP, self-screen prodrome; UHR-PS, ultra high risk psychosis scale; Y-PARQ, youth psychosis at risk questionnaire.
